# Intraoperative Sentinel Lymph Node Evaluation: Implications of Cytokeratin 19 Expression for the Adoption of OSNA in Oral Squamous Cell Carcinoma

**DOI:** 10.1245/s10434-016-5337-6

**Published:** 2016-07-08

**Authors:** Richard Shaw, Anders Christensen, Kapil Java, Rehab El Maddani, Triantafillos Liloglou, Triantafyllou Asterios, Christian von Buchwald, Irene Wessel, Katalin Kiss, Andreas Kjaer, Giedrius Lelkaitis, Anna Long, Janet Risk, Max Robinson

**Affiliations:** 1 Department of Molecular Clinical Cancer Medicine, Mersey Head & Neck Oncology Group, University of Liverpool, Liverpool, UK; 2 Department of Otolaryngology, Head & Neck Surgery and Audiology, Rigshospitalet, Copenhagen University Hospital, Copenhagen, Denmark; 3 Cellular Pathology, Aintree University Hospitals NHS Foundation Trust, Liverpool, UK; 4 Department of Pathology, Rigshospitalet, Copenhagen University Hospital, Copenhagen, Denmark; 5 Department of Clinical Physiology, Nuclear Medicine & PET and Cluster for Molecular Imaging, Rigshospitalet, Copenhagen University Hospital, Copenhagen, Denmark; 6 Cellular Pathology, Newcastle upon Tyne Hospitals NHS Foundation Trust, Newcastle upon Tyne, UK; 7 Centre for Oral Health Research, Newcastle University, Newcastle upon Tyne, UK

## Abstract

**Background:**

Intraoperative analysis of sentinel lymph nodes would enhance the care of early-stage oral squamous cell carcinoma (OSCC). We determined the frequency and extent of cytokeratin 19 (CK19) expression in OSCC primary tumours and surrounding tissues to explore the feasibility of a “clinic-ready” intraoperative diagnostic test (one step nucleic acid amplification—OSNA, sysmex).

**Methods:**

Two cohorts were assembled: cohort 1, OSCC with stage and site that closely match cases suitable for sentinel lymph node biopsy (SLNB); cohort 2, HNSCC with sufficient fresh tumour tissue available for the OSNA assay (>50 mg). CK19 assays included qRT-PCR, RNA in situ hybridisation (ISH), and immunohistochemistry (IHC), as well as OSNA.

**Results:**

CK19 mRNA expression was detected with variable sensitivity, depending on method, in 60–80% of primary OSCC tumours, while protein expression was observed in only 50% of tumours. Discordance between different techniques indicated that OSNA was more sensitive than qRT-PCR or RNA-ISH, which in turn were more sensitive than IHC. OSNA results showed CK19 expression in 80% of primary cases, so if used for diagnosis of lymph node metastasis would lead to a false-negative result in 20% of patients with cervical lymph node metastases.

**Conclusions:**

OSNA in its current form is not suitable for use in OSCC SLNB due to inadequate expression of the CK19 target in all case. However, the same assay technology would likely be very promising if applied using a more ubiquitous squamous epithelial target.

**Electronic supplementary material:**

The online version of this article (doi:10.1245/s10434-016-5337-6) contains supplementary material, which is available to authorized users.

Renewed interest in sentinel lymph node biopsy (SLNB) for early-stage oral squamous cell carcinoma (OSCC) has resulted from reassuring data with 95% negative predictive value (NPV) and also recent trials reinforcing the survival benefit of surgical neck staging.^1^–^3^ A significant drawback of SLNB is that, in the event of a positive lymph node, a costly (and more morbid) second surgical episode is necessitated. This delay, mandated by serial examination of SLN, delays the commencement of adjuvant therapy and creates additional patient distress. SLNB in OSCC would be facilitated by intraoperative staging; however, frozen-section analysis has been found to be somewhat insensitive, certainly highly operator dependent, and has not found general acceptance.^4^–^6^ PCR-based techniques have been reported for head and neck squamous cell carcinoma (HNSCC) but lack a “clinic-ready” platform.^7^,^8^

One-step nucleic acid amplification (OSNA) uses loop-mediated isothermal amplification (LAMP), amplifying RNA with high sensitivity, specificity, efficiency, and rapidity under isothermal conditions.^9^ OSNA employs six specially designed primers at eight sequences within *CK19* mRNA subtending high sensitivity and specificity. In breast cancer, OSNA has been validated to at least 96% concordance with histopathology and has been widely adopted and approved in UK NICE guidelines.^10^,^11^ OSNA necessitates an additional 30–40 min operative time but avoids second surgeries and accelerates commencement of adjuvant therapies from 8.4 to 6.2 weeks.^12^

In HNSCC, the clinical potential of OSNA is unproven, and careful validation is required. Although gene signatures for OSCC or epithelial tissue have been developed with sensitive RT-PCR using other target cytokeratins, or PVA/EPCAM, the opportunity around *CK19* is the availability of a “clinic-ready” diagnostic test with stringent quality assurance.^7^,^8^ Several reports have shown that CK19 is a component of the cytoskeleton of HNSCC and qRT-PCR for cytokeratins appear sensitive and specific in detecting cervical lymph node metastasis in HNSCC.^8^,^13^–^15^*CK19* OSNA has been validated recently for lymph node staging in colorectal and stomach adenocarcinoma.^16^–^19^ The extent of CK19 expression in HNSCC, and therefore whether OSNA could have clinical utility, remains unproven.

Goda et al. analysed 213 HNSCC lymph nodes with CK19 OSNA and suggested an overall accuracy of 94% per node and 94% per patient.^20^ Matsuzuka et al. found a NPV of 95.9% in HNSCC.^21^ Suzuki examined CK19 expression in HSNCC, finding a lower rate of expression and suggesting that clinical use of OSNA only in a selected subset of HNSCC known to be CK19 positive.^22^ All three studies were undertaken in a Japanese population with a variety of stages and sites of HNSCC. For example, Goda et al. report on cT1-4 and N0-3 OSCC and Matsuaka et al. report on a combination of HNSCC sites and also include advanced stages.^20^,^21^ Because SLNB is routinely offered only to cT1-2N0 OSCC, these reports do not ideally reflect the target clinical population in question. It remains uncertain if the expression of CK19 is sufficiently high and uniform to make the CK19 OSNA suitable for use in OSCC SLNB.

The purpose of this study was to establish the frequency and extent of CK19 expression in primary OSCC and surrounding, potentially contaminating, tissues. We established expression of *CK19* mRNA by using both OSNA and other techniques, as well as protein expression. In the event that CK19 expression is <95%, we will pilot assays to be used on diagnostic biopsies of primary tumours to stratify them as suitable, or not, for OSNA analysis of SLNB. Lastly, we tested the concordance between matched primary tumour and metastatic lymph node in CK19 expression.

## Methods

### Tissue

A clinical cohort was assembled from the tissue banks of the Universities of Liverpool and Copenhagen with appropriate ethical approvals and consent. Clinicopathological characteristics and results are summarised in Supplemental Table S1.

Cohort 1 (43 cases from Liverpool) met the criteria: OSCC, clinical stage cT1N0 and cT2N0, formalin-fixed paraffin-embedded (FFPE) and fresh-frozen tumour tissue available. The OSNA assay interrogates fresh (or frozen) sentinel lymph nodes >50 mg, preferably in their entirety. This presented an ethical and logistic barrier as the lymph nodes are required for histopathological staging, and banked primary tumour samples were exclusively <50 mg in T1/T2 OSCC. We therefore elected to analyse primary tumour to establish CK19 expression using a number of assays, *excluding* OSNA. Thirty-four of 43 had available matched FFPE lymph nodes.

Cohort 2 (87 cases: 44 Liverpool and 43 Copenhagen) met the criteria: OSCC, >50 mg snap-frozen primary tumour tissue, most of these were cT3/4 cases.

### Tissue Preparation and Handling

Cohort 1. Immunohistochemistry (IHC): 4-µm sections were stained for CK19 protein by two methods: a mouse monoclonal antibody (clone b170, Leica Biosystems) on a Ventana Benchmark Ultra Autostainer (Ventana Medical Systems, Inc.) at a dilution of 1:100 using standard retrieval conditions (MMC1) and the detection polymer Ultraview (Ventana Medical Systems, Inc.): a mouse monoclonal antibody (clone RCK 108, Dako) diluted 1:100 and the EnVision FLEX system on an Autostainer Link 48 instrument (Dako) using high pH antigen retrieval. Negative controls omitted addition of the primary antibody.

In situ hybridization (ISH): *CK19* RNA ISH was performed on 4-μm FFPE sections using proprietary reagents (RNAscope, Advanced Cell Diagnostics, Inc.). Sections were deparaffinised and pretreated with heat and protease before hybridisation with target-specific probes: *CK19*, *PPIB* (constitutively expressed endogenous gene; positive control) and *dapB* (bacterial mRNA; negative control) in a dedicated hybridization oven (HybEZ oven, Advanced Cell Diagnostics, Inc.). Probe hybridization was detected using the chromogen 3,3′-diaminobenzidine (DAB).

Both IHC and ISH techniques were optimized using known positive (breast ductal carcinoma) and negative tissue (lymph node). Tissue cores from controls constituted a “control block”—sections of which were mounted on each test slide to ensure quality staining methods. The tests were scored by two pathologists (MR & AT). Staining was assessed by assigning an intensity score (0, no staining; 1, weak; 2, moderate; 3, strong) and percentage of malignant cells stained. These were used to calculate an H score (product of intensity and percentage) but also classified in a binary fashion (positive vs. negative).

RNA was prepared from fresh-frozen tissue of primary tumours using an miRNeasy kit (Qiagen), and following reverse transcription (cDNA kit, Applied Biosystems), a CK19 qRT-PCR assay was performed with the following primers/probe; Fwd: 5′CACTACTACACGACCATCCAGGAC 3′, Rev: 5′ CGGAAGTCATCTGCAGCCA 3′, Probe: 5′ TAMRA-ACGGGCATTGTCGATCTGCAGGAC-BHQ2. The qPCR reaction utilised the Universal Master Mix II (Applied Biosystems), the thermal profile: 50 °C for 2 min, 95 °C for 10 min, 45 cycles of 95 °C for 15 sec, and 60 °C for 1 min, using a 7500 FAST instrument (Applied Biosystems). The relative quantification (RQ) value was calculated as: RQ = 2^−∆∆Ct,^ where Ct is the cycle threshold for each target.

Cohort 2. OSNA: OSCC biopsies from cohort 2 with mass between 50 and 600 mg were snap frozen and stored at −80 °C until shipment to Sysmex on dry ice. Samples were processed according to manufacturer’s instructions (Sysmex, Kobe, Japan) using a designated instrument (RD-100*i*) and reagent system (LYNOAMP & LYNORHAG). Individual tumour samples were placed in 4 ml of homogenizing buffer LYNORHAG (0.2 M glycine-HCl pH 3.5, 5% Brij35 and 20% DMSO), and homogenised for 60 s at 10,000 rpm with a Polytron System PT1300D (Kinematica AG, Switzerland) and LYNOPREP blades to prepare a homogeneous lysate. One milliliter of lysate was centrifuged to remove cell debris and then further diluted 1:10 and 1:100 with LYNORHAG. The diluted lysates were used directly for amplification without RNA extraction or purification. Isothermal amplification reactions were performed at 65 °C. The rise time required for precipitation of magnesium pyrophosphate to reach a turbidity of 0.1 OD at 465 nm was obtained for each sample and the number of *CK19* mRNA copies determined using a calibration curve. OSNA was classified as following: (−) = <250 copies; (−L) = <250 copies; (+) = >250 and <5000 copies, (++) = > 5000 copies; (++) or (+) were positive results, whereas (−) or (−L) were negative.

RNA quality was analysed for negative (− or −L) samples to exclude false negatives. OSNA lysates were processed with the Qiagen RNeasy kit (Qiagen, Venlo, Netherlands). Total RNA was quantified spectrophotometrically (260/280 nm ratio). RNA integrity was assessed using RNA Integrity Number (RIN) with a Bioanalyser (Agilent, Santa Clara, CA).

RNA was prepared from unused OSNA lysates and from a separate aliquot of fresh-frozen tissue from the same tumours, reverse transcribed and subject to *CK19* qRT-PCR assay as described above.

Interplate qRT-PCR variation was reduced by using the ΔΔCt method to normalise expression with respect to two tumours that had previously been shown to highly express CK19. A technical threshold of 0.005 × the mean ΔCt of the reference tumours was observed in two experiments and was adopted to distinguish positive from negative *CK19* expression in all qRT-PCR experiments.

## Results

**Fig. 1:**
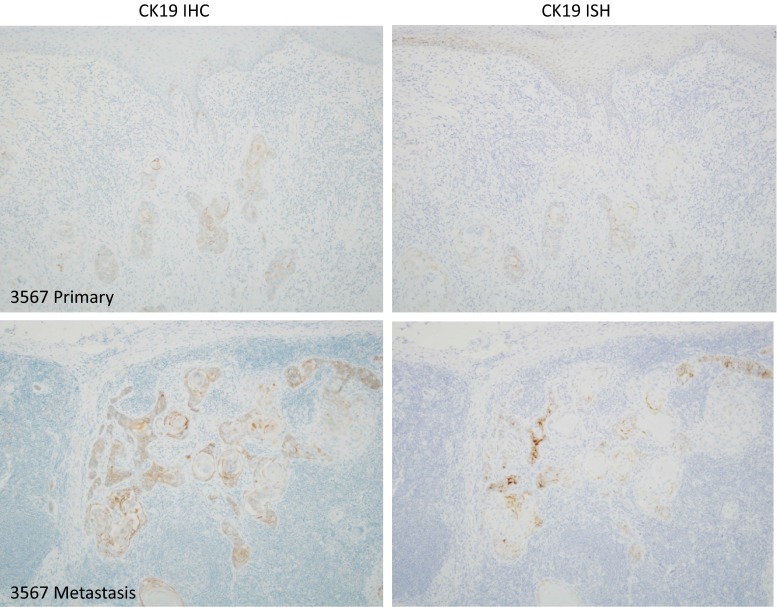
A primary tumour that shows weak, heterogeneous CK19 positivity, and a corresponding subcapsular lymph node metastasis with stronger CK19 staining. This case illustrates the difficulty, with either IHC or ISH, to offer a confident diagnostic test to identify cases from diagnostic biopsy suitable for CK19 OSNA

**Fig. 2:**
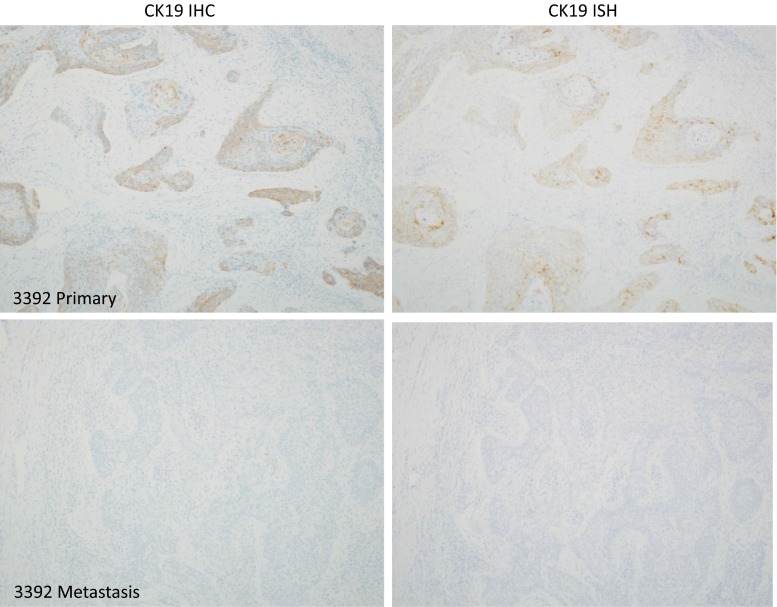
A primary tumour that shows weak, heterogeneous CK19 positivity, and a corresponding lymph node metastasis with no CK19 staining. If this case had undergone SLNB analysis using CK19 OSNA, even with the apparent security of a “positive” primary tumour, it is likely that a false-negative result would be returned with consequent undertreatment and neck recurrence

Cohort 1. Of 43 primary cT1/T2N0 tumours tested with CK19 IHC, 21 (48.8%) were positive. Twenty-nine of 39 primary tumours evaluable in *CK19* RNA ISH tests were *CK19* positive (74.4%); 4 failed quality assurance checks. CK19 IHC was concordant with the *CK19* RNA ISH in 26 of 39 cases (66.7%). For both tests, the staining was generally weak and heterogeneous (Figs. 1, 2) with positive cases having H scores between 5 to 200. Discordant cases (*n* = 13) had lower H scores (mean 42.7; range 5–160). Of the 13 discordant results, 9 were positive in RNA ISH and negative in IHC, reflecting higher sensitivity of RNA ISH. Stage I/II OSCC was less likely to be positive by IHC than stage III/IV (*P* < 0.01). No such discrepancy was observed for RNA ISH.

CK19 IHC and *CK19* RNA ISH were concordant in 6 of 8 cases with corresponding lymph node metastases, omitting two failed tests (Table 1). In one case, the primary tumour was positive for both tests, but the corresponding lymph node metastasis was negative (patient 3392; Fig. 2; Table 1). Lymph nodes with no evidence of metastatic carcinoma (*n* = 26) did not contain any CK19-positive cells. There were no epithelial lymph node inclusions (salivary or thyroid); however, in one case CK19 positive perinodal salivary gland tissue was included in the section, but this might have been dissected free prior to analysis in an SLNB protocol.

**Table 1:** Test results for primary tumours with corresponding lymph node metastases

*Red* positive,* blue* negative,* yellow* not available, test failed quality assurance checks

Eighteen of 26 (69%) primary cT1/T2N0 tumour tissues were positive for *CK19* mRNA by qRT-PCR. *CK19* qRT-PCR was concordant with IHC in 16 of 26 cases (62%) and with *CK19* RNA ISH in 13 of 22 cases (59%; Table 2). Discordant IHC cases tended to be positive by qRT-PCR (7/10). By contrast, discordant ISH cases were equally likely to be positive or negative (4/9 positive by qRT-PCR); however, the ISH positives had lower H scores (mean 33.0, range 5–160).

**Table 2:** qRT-PCR concordance with IHC and ISH staining

		IHC		ISH	
		+	−	+	−
qRT-PCR	+	11	7	11	4
qRT-PCR	−	3	5	5	2

Cohort 2. Of 87 primary tumour samples analysed by OSNA (Table 3), 7 were excluded because of compromised RNA integrity (low RIN). Examination of representative, H&E stained sections from the 43 Danish samples identified 5 that did not contain tumour tissue by pathological examination and 1 with compromised RNA integrity. The remaining 4 were OSNA positive: 2 contained oral epithelium and 2 contained salivary tissue. Of 76 tumour samples, 61 (80%) were *CK19* mRNA positive by OSNA, with no correlation for either tumour stage or site (Supplementary Table S2).

**Table 3:** Distribution of *CK19* mRNA expression by OSNA

OSNA	No. of samples (%)	%
**(++)**	45 (56)	65 (81)
**(+)**	20 (25)	
**(−)**	12 (15)	15 (19)
(−L)	3 (4)	
Total	80 (100)	

Thirty-nine of the tumours from Liverpool had sufficient tissue to allow extraction of mRNA from a separate portion of the tumour. Of these, 23 (59%) were *CK19* positive. qRT-PCR data was concordant with OSNA data in 29 of 37 (78%) of cases, with OSNA proving the most sensitive test in all 8 discordant cases. To investigate this more fully, RNA from the OSNA tissue lysates from all 87 samples was subject to qRT-PCR. Four samples were excluded based on the low RNA levels (GAPDH amplification). Of these samples, 56 of 83 (67%) were positive for *CK19* expression, showing concordance with OSNA data in 70 of 81 (85%) of cases. All discordant cases demonstrated positivity by OSNA but were negative by qRT-PCR.

## Conclusions

CK19 expression is detectable for 50–80% of OSCC, depending upon the assay used. RNA-ISH and qRT-PCR are more sensitive than IHC, whereas OSNA appears to be the most sensitive method. The prevalence of *CK19* expression by OSNA is still, at 80%, insufficient to suggest that OSNA could be used without prior screening of biopsy tissue for CK19, because it could result in 20% of positive lymph nodes being called as false-negative. CK19 expression in OSCC has been reported previously to range from 53% to 91%.^13^,^14^ Our results confirm that CK19 can be detected only in a subset of primary tumours. In this regard, OSCC differs from breast, colorectal and stomach sites, all adenocarcinomas, where CK19 OSNA has been clinically validated. Although the chemistry and platform available through OSNA appear to be well suited to clinical use in being highly reliable, sensitive, and specific, the gene target CK19 appears to offer insufficient expression in OSCC for clinical application. Should a more appropriate gene target (perhaps CK5 or 14) be available, it may be that this would be suitable, subject to the appropriate and necessary clinical validations.

Although, theoretically, OSNA might be used on a fresh biopsy sample to select CK19 positive tumours suitable for OSNA assay in SLNB, concern remains that surrounding oral mucosa or salivary gland could be included leading to a false positive. *CK19* mRNA ISH performed on an existing FFPE diagnostic biopsy might be more convenient and provide histological context, avoiding false positives. However, our results show that CK*19* mRNA ISH expression was usually low and heterogeneous, limiting diagnostic confidence and making the assay vulnerable to interobserver variability. Consequently, we could not suggest a reliable assay to stratify which tumours are suitable for OSNA assay in SLNB.

In one case, the primary tumour was positive and the matched lymph node metastasis was negative by both CK19 ISH and IHC. Contamination in the neck structures with ectopic salivary (0.9%) or thyroid tissue (1.5%) have been reported either within or immediately surrounding lymph nodes and could produce false positives in any methodology that uses solid specimens.^23^,^24^ It may be that careful dissection of single SLNB would eliminate this, but again a validation study would be helpful.

Our data successfully incorporated a new assay (*CK19* mRNA ISH) and shows potential clinical avenues in OSCC for molecular diagnostics. We have *CK19* data on 123 OSCC that effectively rules out the need for potentially burdensome, and clinically risky, validation studies. The international collaboration between two academic head and neck cancer centres and industry augers well should a more suitable assay become available. Such an assay might additionally be applicable to cutaneous SCC and anogenital SCC, which would increase the test’s commercial viability. It is encouraging that OSNA assays with differing gene targets, most recently with MMP7 (matrix-metalloproteinase 7) are available.^25^

The concept of intraoperative diagnostics in OSCC remains attractive but awaits a suitable assay. At present, SLNB analysis is based on evaluation of stepped serial sections from only a proportion of the sentinel node, thus a rapid technique examining the entire sentinel node for tumour deposits may provide more accurate staging. An automated intraoperative method also would avoid the substantial additional workload for the pathology team performing serial SLNB examination. In head and neck oncology, intraoperative diagnostics appear even more attractive than in melanoma and breast, because OSCC remains largely a surgically treated disease and completing all surgery in one operation would facilitate the wider acceptance of SLNB.

## Electronic Supplementary Material

Below is the link to the electronic supplementary material.

Supplementary material 1 (XLSX 39 kb)

Supplementary material 2 (DOCX 31 kb)
