# Supplementary material for: Intraoperative Sentinel Lymph Node Evaluation: Implications of Cytokeratin 19 Expression for the Adoption of OSNA in Oral Squamous Cell Carcinoma
Source: Ann Surg Oncol. 2016 Jul 8;23(12):4042–8. doi: 10.1245/s10434-016-5337-6 (PMC5047925; doi:10.1245/s10434-016-5337-6)
Supplement: Supplementary file 2 — Supplementary material 2 (DOCX 31 kb) [file 10434_2016_5337_MOESM2_ESM.docx]

Supplementary Table 2. Distribution of *CK19* expression by OSNA and stage

|  | | OSNA | |
| --- | --- | --- | --- |
|  |  | + | - |
| Stage II |  | 4 | 1 |
| Stage III |  | 30 | 6 |
| Stage IV |  | 30 | 6 |
